# Use of Disopyramide in Obstructive Hypertrophic Cardiomyopathy: A European Insight

**DOI:** 10.3390/jcm15114234

**Published:** 2026-05-30

**Authors:** Philippe Charron, Faizel Osman, Jean-Noel Trochu, Carla Zema, Michael Hurst, Belinda Sandler, François-Emery Cotté, Teresa Lemmer, Maite Tome Esteban

**Affiliations:** 1 Department of Cardiology & Department of Genetics, Hôpital Universitaire Pitié-Salpêtrière, IHU-ICAN, INSERM 1166, AP-HP, Sorbonne Université, 75013 Paris, France; 2 Institute of Cardio-Metabolic Medicine, University Hospitals Coventry and Warwickshire NHS Trust, Coventry CV2 2DX, UK; 3 Warwick Medical School, University of Warwick, Coventry CV4 7AL, UK; 4 L’institut du Thorax, Hôpital Universitaire, Nantes Université, INSERM, Centre National de Recherche Scientifique, 44035 Nantes, France; 5 Bristol Myers Squibb, Princeton, NJ 08543, USA; 6 Bristol Myers Squibb, Uxbridge UB8 1DH, UK; 7 Bristol Myers Squibb, 92506 Rueil-Malmaison, France; 8 Department of Cardiology, St George’s NHS Foundation Trust & St George’s University of London, London SW17 0QT, UK

**Keywords:** disopyramide, guideline-directed treatment, hypertrophic cardiomyopathy, left ventricular outflow tract obstruction, obstruction, therapy

## Abstract

**Background/Objectives:** Guidelines for obstructive hypertrophic cardiomyopathy (HCM) recommend treatment with disopyramide as an add-on to beta-blockers or calcium-channel blockers when symptoms persist. Data pertaining to effective disopyramide use in practice beyond single-center experience are very limited. This study aimed to quantify disopyramide use in patients with obstructive HCM in England, France and Germany, before the availability of cardiac myosin inhibitors. **Methods:** This retrospective study used nationally representative databases from England (Clinical Practice Research Datalink and Hospital Episode Statistics, 2010–2019), France (National Healthcare Data System, 2012–2019) and Germany (German statutory health insurance, 2011–2019). Adults (18+) with obstructive HCM were included, based on diagnostic codes for obstructive HCM or any HCM with septal reduction therapy. Disopyramide usage was defined as ≥1 prescription for a patient in a calendar year. **Results:** Overall, 3730, 6823 and 1141 patients diagnosed with obstructive HCM were identified in the English, French and German databases, respectively. In England, disopyramide use ranged from 4.7% to 5.6% per year with use generally stable over time. The equivalent usage for France was 1.7% to 2.6% per year. As expected, no recorded reimbursed use was reported in Germany during the study period. **Conclusions:** Disopyramide use is very low in patients with obstructive HCM, possibly due to treatment-related issues, availability or lack of reimbursement. These barriers may drive the uptake of alternative guideline recommended therapies for obstructive HCM treatment.

## 1. Introduction

Hypertrophic cardiomyopathy (HCM) can be genetic and is the most common inherited cardiac disorder; it is characterized by left ventricular hypertrophy not explained by abnormal loading conditions [1,2,3,4]. Approximately 70% of patients with HCM have obstructive HCM, which is defined as an obstruction in the left ventricular outflow tract (LVOT) either at rest or with provocation, with patients experiencing varying symptomatic burden including dyspnea, chest pain, fatigue, palpitations and syncope [1,2].

European Society of Cardiology (ESC) and American Heart Association/American College of Cardiology (AHA/ACC) guidelines for the treatment of symptomatic obstructive HCM advise the addition of disopyramide for patients who have persistent symptoms despite use of non-vasodilating beta-blockers (BBs) or non-dihydropyridine calcium-channel blockers (CCBs) [3,4]. The guidelines recommend disopyramide use in these patients at an equivalent or higher recommendation class level than cardiac myosin inhibitor therapy; however, the precise use of disopyramide in practice at a national level has not been previously assessed [3,4]. A study in a large specialty hospital found that 41% of patients with obstructive HCM did not respond to first-line therapy with BBs or CCBs [5]. This study also investigated disopyramide use among patients who required further therapy and provided the primary evidence to support HCM guidance regarding disopyramide therapy for the treatment of advanced obstructive HCM [3,4,5]. Despite the potential for disopyramide to reduce the LVOT gradient owing to its negative inotropic properties and augmentation of systemic vascular resistance, use of disopyramide may be limited owing to the associated rates of non-response (up to one-third), availability, side effect tolerance and discrete QT prolongation [5,6,7].

Variation can be seen in reimbursement policies for disopyramide across different countries. Although disopyramide is fully reimbursed and low cost in many countries, access varies across Europe. Disopyramide has never been reimbursed for use in Germany, is available in France despite an unfavorable reimbursement opinion (with reported supply issues), and is currently subject to a medicine supply notification in England due to intermittent stock availability [8,9,10].

It is important to understand disopyramide use in a large population, as quantifying real-world prescribing patterns may help identify potential gaps between guideline recommendations and clinical practice, as well as regional differences in access and uptake. Therefore, the objective of this analysis was to quantify use of disopyramide through analysis of nationwide databases from three countries.

## 2. Methods

### 2.1. Data Sources

This retrospective study used nationwide or nationally representative databases from three countries: Clinical Practice Research Datalink (CPRD) primary care data linked with Hospital Episode Statistics secondary care data in England from 2010 to 2019; French National Healthcare Data System (SNDS) data from 2012 to 2019; and Wissenschaftliches Institut für Gesundheitsökonomie und Gesundheitssystemforschung (WIG2) benchmark database of several German statutory health insurances from 2011 to 2019 (Table 1).

### 2.2. Study Population

Eligible patients were adults (≥18 years of age) with a diagnosis of obstructive HCM, based on an obstructive HCM diagnosis or diagnosis of any HCM in the presence of a septal reduction therapy procedure. All relevant coding systems applicable to the specific country were used (e.g., International Classification of Diseases, Tenth Revision, Clinical Modification; Read codes; and Systematized Nomenclature of Medicine Clinical Terms codes). The diagnosis, procedure and prescription codes used in the analysis are summarized in the Supplementary Materials (Tables S1 and S2).

### 2.3. Outcomes

Patient characteristics were assessed either at or up to 2 years before the first obstructive HCM diagnosis during the study period. Disopyramide use was based on the number of patients with obstructive HCM and at least one disopyramide prescription in a calendar year. There were two clinical HCM guidelines in effect during the enrollment period—the 2003 guidelines and the 2014 guidelines. Per the 2003 ACC/ESC guidelines [11], disopyramide was considered a favorable pharmacologic therapy, typically reserved for patients with obstructive HCM who were severely symptomatic. It was often administered in combination with BBs, although co-administration with verapamil was discouraged. Following the publication of the 2014 ESC guidelines [12], patients could receive disopyramide if symptomatic with resting or provocable LVOT obstruction, with treatment titrated to the maximum tolerated dose and used either in combination with BBs or verapamil or as monotherapy. To account for the potential use of unspecified HCM codes rather than obstructive HCM diagnosis codes, the percentage of all patients with HCM with at least one prescription of disopyramide was also calculated.

### 2.4. Ethics Approval

This study complies with the principles outlined in the Declaration of Helsinki. The research protocol was approved by the respective locally appointed ethics committees (aligned with the respective guidance for utilizing each of the datasets: England, Independent Scientific Advisory Committee application reference 21_000342; France, Comité éthique et scientifique pour les recherches, les études et les évaluations dans le domaine de la santé file 1912776bis), and permission was granted by the database owners to use the data. The use of the anonymized WIG2 database for scientific purposes is in conformity with German law; as such, no ethics committee approval was required. Only aggregated data are presented in this study, and the patient-level data used were pseudo-anonymized to reflect the appropriate requirements for accessing the aggregated datasets. As these data were used retrospectively, written consent was not required.

## 3. Results

In total, 6440 unique patients with HCM (3730 with obstructive HCM) were identified from 2010 to 2019 in the English CPRD database, 20,111 patients (6823 with obstructive HCM) from 2012 to 2019 in the French SNDS database, and 2601 patients (1141 with obstructive HCM) from 2011 to 2019 in the German WIG2 database.

### 3.1. Patient Characteristics

Of the patients with obstructive HCM (Table 2), 39%, 45% and 38% were female in England, France and Germany, respectively. Mean (95% confidence interval) age at the first obstructive HCM diagnosis in the study period was 61.2 (60.7, 61.7), 65.5 (65.1, 65.9) and 59.6 (58.6, 60.6) years in each of the respective countries. Common cardiovascular comorbidities were similar across the countries; the majority had primary hypertension during their baseline period. Within each of the three countries, the proportion of the obstructive HCM population independent of follow-up time with a prescription for disopyramide use was very low (England: 7.6%; France: 3.3%) or, in the case of Germany, was zero. In England and France, where disopyramide usage was recorded, patients with obstructive HCM receiving disopyramide were younger on average and had fewer comorbidities (particularly atrial fibrillation/flutter), represented by a mean Charlson comorbidity score of 2.37 (vs. 3.01) in England and 2.15 (vs. 3.90) in France when compared with the patient population who did not receive disopyramide.

### 3.2. Prevalence of Disopyramide Use

**Figure 1:**
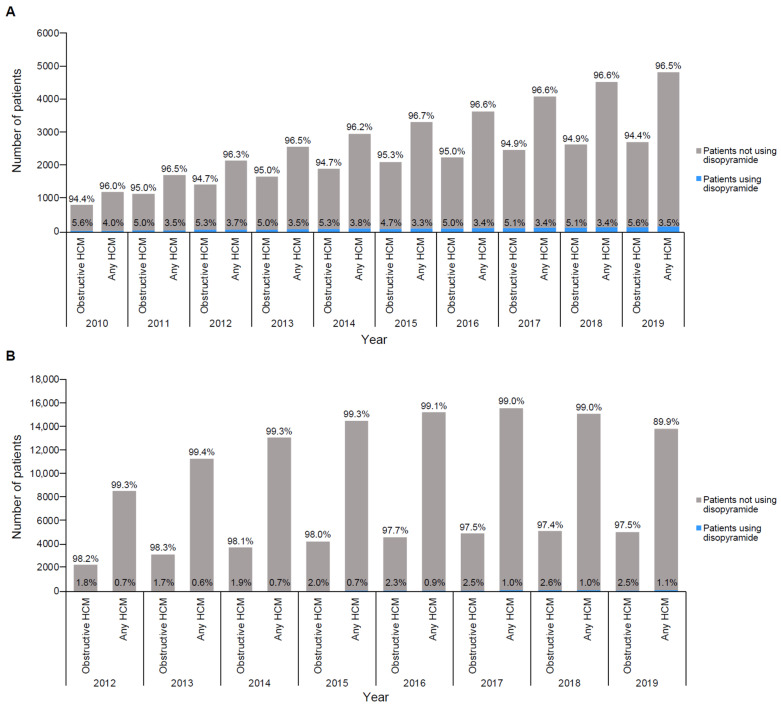
Disopyramide use each year in England (**A**) and France (**B**). No reimbursed disopyramide use was confirmed in Germany from 2011 to 2019. HCM—hypertrophic cardiomyopathy.

The percentage of patients with obstructive HCM who received disopyramide in a given calendar year was generally stable from 2010 to 2019 in England and ranged from 4.7% to 5.6% per year (Figure 1). Very few additional patients receiving disopyramide were added when analyzing use among all patients with HCM. In France, disopyramide use in patients with obstructive HCM was lower than in England and ranged from 1.7% to 2.6% per year, with prevalence generally increasing from 2012 to 2019. Although additional patients receiving disopyramide were identified when analyzing all patients with HCM, usage remained extremely low. No reimbursed disopyramide use was recorded in Germany from 2011 to 2019 within the obstructive HCM cohort.

## 4. Discussion

This is the first study to examine disopyramide use among populations of adult patients with obstructive HCM in multiple countries in Europe. The datasets for England and France are largely representative of the respective overall adult populations, and the large dataset for Germany has been shown to reflect the population covered by statutory health insurance [13]. Across these settings, disopyramide use was consistently low, with a maximum yearly prevalence of 5.6% in England, 2.6% in France, and no recorded reimbursed use in Germany despite guideline recommendations. In countries where disopyramide use was observed, treatment was more frequently prescribed in younger patients with fewer comorbidities. These findings highlight a gap between guideline recommendations and real-world clinical practice and demonstrate variation in treatment patterns across healthcare systems, providing important context for understanding management of obstructive HCM in routine care in the time period observed.

To date, no phase 3 randomized controlled trial has been performed to assess the efficacy and safety of disopyramide treatment in patients with obstructive HCM. However, multiple observational studies have demonstrated that disopyramide treatment (often in conjunction with BBs or CCBs) is associated with substantial improvements in resting LVOT gradient and New York Heart Association (NYHA) functional class [5,14,15]. Despite this, and despite disopyramide being guideline-recommended for the treatment of patients with obstructive HCM who have not responded to BB/CCB therapy, extremely low use was observed among patients with obstructive HCM. This result might be related to insufficient awareness in the medical community about the potential benefit of this drug. In addition, disopyramide prescribing may be concentrated in specialist HCM centers and therefore underrepresented in population-based datasets. This result may reflect several factors, including differences in access, reimbursement, and drug availability across healthcare systems. Alternatively, the findings of the present study may reflect treatment-related factors, including variability in response, issues with patient adherence and potential side effects with disopyramide, including corrected QT interval prolongation and anticholinergic effects [5,6,7,15]. With the inclusion of newer drugs within most recent treatment guideline updates, disopyramide may be less frequently used in the treatment of arrhythmias regardless of the presence of HCM. Furthermore, the ESC 2020 Guidelines for the diagnosis and management of atrial fibrillation include messages about the need to exercise caution when using Class Ia antiarrhythmic drugs (i.e., quinidine and disopyramide), which have been associated with increased overall mortality [16,17]. In some countries, limited use of disopyramide is likely to be related to a lack of reimbursement and market approval. Some countries have never had disopyramide available, have an unfavorable clinical assessment of it, or are facing shortages [8,10].

Anecdotal evidence from practicing clinicians suggests that only specialized referral centers that treat patients with advanced HCM use disopyramide. Although these highly specialized centers may use disopyramide more frequently than non-specialized centers, the population estimates illustrate that very few patients with obstructive HCM receive disopyramide each year. This observation is supported by the findings of VALOR-HCM, a US study which was designed to determine whether mavacamten therapy (a selective, cardiac-specific myosin inhibitor for patients with symptomatic obstructive HCM) would enable patients to improve sufficiently to no longer require, or be guideline eligible for, septal reduction therapy. In VALOR-HCM, 25.0% and 14.3% of patients in the mavacamten and placebo groups, respectively, were receiving disopyramide as monotherapy or alongside other therapies at baseline [18]; highlighting that, even in patients with severely symptomatic obstructive HCM, disopyramide use is limited. The updated guidelines for the management of hypertrophic cardiomyopathy from the ESC 2023 and the American Heart Association/American College of Cardiology Joint Committee on Clinical Practice Guidelines 2024, still recommend disopyramide use (Class I/Level B) but now along with the recently approved mavacamten (Class IIa/Level A) [3,4].

A limitation of this analysis is the potential miscoding of obstructive HCM. ‘Unspecified’ HCM is an option in diagnosis coding; however, when disopyramide use was examined across a wider population of patients that included those with ‘unspecified HCM’, very few additional disopyramide users were identified. In addition, the use of diagnostic codes may not fully distinguish sarcomeric HCM from phenocopies (e.g., Fabry disease, cardiac amyloidosis, or glycogen storage disorders), which differ in underlying pathophysiology and management and may influence prescribing patterns [19]. Of course, under-recognition of HCM is also known to be problematic, which leads to patients not receiving the appropriate diagnosis. Since patients are prescribed disopyramide following diagnosis of HCM, misdiagnosis of HCM is only likely to reduce the prevalence of disopyramide treatment. Disopyramide usage was defined as a patient with one or more prescriptions within a calendar year. As such, values could be seen as an overestimate of true prevalence, as it fails to consider the duration of treatment, dosing adherence and dosing persistence. In addition, the denominator used in the analysis included all patients with obstructive HCM, irrespective of symptomatic status or prior treatment exposure. This approach may have underestimated the proportion of patients eligible for disopyramide according to guideline criteria. Finally, the absence of linked clinical outcomes data precludes assessment of whether the observed low use of disopyramide is associated with suboptimal patient outcomes.

Patients receiving disopyramide were younger and had fewer comorbidities, which may reflect selective prescribing based on clinical risk and contraindications rather than random treatment allocation. This potential bias should be considered when interpreting treatment patterns. Further research of the geographical variation in disopyramide use, both within countries and across countries, and use by NYHA functional class may be helpful to improve our understanding of the variations in disopyramide use in Europe.

To conclude, disopyramide use among patients with obstructive HCM was found to be consistently low in England between 2010 and 2019 and in France between 2012 and 2019. As expected, no reimbursed disopyramide use was identified in Germany between 2011 and 2019. Despite previous studies suggesting that a substantial proportion of patients with obstructive HCM who receive BBs or CCBs are eligible for treatment intensification with disopyramide [5], use of the drug remained minimal across countries. These findings reflect treatment patterns prior to the introduction of cardiac myosin adenosine triphosphatase inhibitors [20,21] and should be interpreted within the context of evolving guideline-recommended therapies for obstructive HCM.

## Figures and Tables

**Table 1:** Comparison of data sources.

Feature	England (CPRD-HES)	France (SNDS)	Germany (WIG2)
Population coverage	Primary care linked data	National claims database	Statutory insurance sample
Study period	2010–2019	2012–2019	2011–2019
Prescription capture	GP prescriptions	Reimbursed claims	Insurance claims

CPRD—Clinical Practice Research Datalink; GP—general practice; SNDS—French National Healthcare Data System; WIG2—Wissenschaftliches Institut für Gesundheitsökonomie und Gesundheitssystemforschung.

**Table 2:** Baseline characteristics of patients with obstructive HCM.

Characteristics	England			France			Germany
	Overall obstructive HCM (*n* = 3730)	Disopyramide use (*n* = 284)	No disopyramide use (*n* = 3446)	Overall obstructive HCM (*n* = 6823)	Disopyramide use (*n* = 227)	No disopyramide use (*n* = 6596)	Overall obstructive HCM (*n* = 1141)
Female, *n* (%)	1473 (39.5)	142 (50.0)	1331 (38.6)	3093 (45.3)	107 (47.1)	2986 (45.3)	430 (37.7)
Age, years, mean (95% CI)	61.2 (60.7, 61.7)	58.1 (56.5, 59.7)	61.4 (60.9, 61.9)	65.5 (65.1, 65.9)	54.1 (52.2, 56.0)	65.9 (65.5, 66.3)	59.6 (58.6, 60.6)
CCI score, mean (95% CI)	2.96 (2.88, 3.04)	2.37 (2.13, 2.61)	3.01 (2.93, 3.09)	3.84 (3.78, 3.90)	2.15 (1.94, 2.36)	3.90 (3.84, 3.96)	2.34 (2.21, 2.47)
Comorbidities, *n* (%)							
Atrial fibrillation/flutter	574 (15.4)	27 (9.5)	547 (15.9)	1876 (27.5)	37 (16.3)	1839 (27.9)	172 (15.1)
DVT/PE	56 (1.5)	NR	NR	123 (1.8)	1 (0.4)	122 (1.9)	34 (3.0)
Heart failure	564 (15.1)	32 (11.3)	532 (15.4)	1674 (24.5)	40 (17.6)	1634 (24.8)	246 (21.6)
Hypertension	1806 (48.4)	126 (44.4)	1680 (48.8)	5869 (86.0)	194 (85.5)	5675 (86.0)	727 (63.7)
Myocardial infarction	297 (8.0)	15 (5.3)	282 (8.2)	170 (2.5)	2 (0.9)	168 (2.5)	50 (4.4)
Stroke/TIA	405 (10.9)	20 (7.0)	385 (11.2)	417 (6.1)	8 (3.5)	409 (6.2)	102 (8.9)
Previous septal reduction therapy, *n* (%)	6 (0.2)	NR	NR	18 (0.3)	0 (0.0)	18 (0.3)	10 (0.9)

CCI—Charlson Comorbidity Index; CI—confidence interval; DVT—deep vein thrombosis; HCM—hypertrophic cardiomyopathy; NR—not reported due to small numbers; PE—pulmonary embolism; TIA—transient ischemic attack.

## Data Availability

The datasets generated during and/or analyzed during the current study are not publicly available and only available upon license for the respective data custodians within each of the three countries involved in this study. Further information for data access for CPRD can be found at: https://www.cprd.com/data-access (accessed on 19 April 2026); SNDS at https://www.snds.gouv.fr/SNDS/Accueil (accessed on 19 April 2026); WIG2 at https://www.wig2.de/diga-digital-health-applications.html (accessed on 19 April 2026).

## Supplementary Materials

The following supporting information can be downloaded at: https://www.mdpi.com/article/10.3390/jcm15114234/s1, Table S1: Diagnostic and procedure codes; Table S2: Disopyramide prescription codes.

## Abbreviations

The following abbreviations are used in this manuscript:

ACC	American College of Cardiology
AHA	American Heart Association
BB	Beta-blocker
CCB	Calcium-channel blocker
CCI	Charlson Comorbidity Index
CI	Confidence interval
CPRD	Clinical Practice Research Datalink
ESC	European Society of Cardiology
HCM	Hypertrophic cardiomyopathy
LVOT	Left ventricular outflow tract
NYHA	New York Heart Association (functional class)
PE	Pulmonary embolism
SNDS	French National Healthcare Data System
TIA	Transient ischemic attack
WIG2	Wissenschaftliches Institut für Gesundheitsökonomie und Gesundheitssystemforschung
