# Supplementary material for: Use of Disopyramide in Obstructive Hypertrophic Cardiomyopathy: A European Insight
Source: J Clin Med. 2026 May 30;15(11):4234. doi: 10.3390/jcm15114234 (PMC13258706; doi:10.3390/jcm15114234)
Supplement: Supplementary file 1 [file jcm-15-04234-s001.zip › jcm-4320028-supplementary.pdf]

Supplementary Materials

SUPPLEMENTAL TABLE S1 Diagnostic and procedure codes

Condition	ICD-10	OPCS	SNOMED CT	Read code	Med code
HCM	I42.1, I42.2		233873004, 195020003, 198381000000104, 45227007	Gyu5M, ^ESCTHC505683, ^ESCTHY477709, G5543, 12CR, ^ESCTHO322942, G551, ^ESCTFA805828, ^ESCTHC477711, 12CR.00, G551.00, G554300, Gyu5M00	300910013, 5056831000006119, 4777091000006118, 300038015, 298941000000114, 3229421000006114, 75410014, 8058281000006111, 4777111000006110, 42999, 8010, 3499, 70648
LVOT obstruction		K24.5, K24.6, K24.7	253546004	P69, ^ESCTLV533146, ESCTLE1, P69..00	1908831000006118, 5331461000006116, 377550012, 108457
Non-obstructive HCM	I42.2		195020003	^ESCTHY477709, G5543, ^ESCTHC477711, G554300	4777091000006118, 300038015, 4777111000006110, 3499
Obstructive HCM	I42.1		198381000000104, 45227007	12CR, ^ESCTHO322942, G551, ^ESCTFA805828, 12CR.00, G551.00	298941000000114, 3229421000006114, 75410014, 8058281000006111, 42999, 8010
Septal reduction therapy		K16.6	223341000000109, 174997007, 428553005, 233091008, 437746009	790N4, ^ESCTOP466510, ^ESCTLV466512, 790N5, ^ESCTEX709017, 790N6, 790C5, ^ESCTAL719940,	355821000000117, 4665101000006118, 4665121000006111, 399361000000112, 7090171000006115, 1546951000006118, 357821000000110,

				^ESCT1270514,<br>^ESCTPE719939,<br>790N500, 790N600,<br>790C500, 790N400	7199401000006117,<br>12705141000006118,<br>7199391000006119, 90112,<br>114629, 90998, 93408

HCM, hypertrophic cardiomyopathy; ICD-10, International Classification of Diseases, Tenth Revision; LVOT, left ventricular outflow tract; OPCS, Office of Population Censuses and Surveys Classification of Interventions and Procedures; SNOMED CT, Systematized Nomenclature of Medicine Clinical Terms

SUPPLEMENTAL TABLE S2 Disopyramide prescription codes

Product name	DMD code	BNF code
Disopyramide 100mg capsules	3465211000001104	02030201
Disopyramide 100mg capsules	318259005	02030201
Disopyramide 100mg capsules	3463911000001107	02030201
Disopyramide 25mg/5ml oral solution	8454711000001102	02030201
Disopyramide 50mg/5ml oral solution	8454511000001107	02030201
Disopyramide 150mg capsules	318260000	02030201
Disopyramide 150mg capsules	3734711000001106	02030201
Disopyramide 100mg/5ml oral suspension	8454811000001105	02030201
Disopyramide 20mg/5ml oral solution	12081011000001105	02030201
Disopyramide 250mg tablets	3692111000001102	02030201
Disopyramide 50mg/5ml solution for injection ampoules	4867311000001109	02030201
Disopyramide 250mg modified-release tablets	36070311000001106	02030201

BNF, British National Formulary; DMD, dictionary of medicines and devices
